# Predictive value of different bilirubin subtypes for clinical outcomes in patients with acute ischemic stroke receiving thrombolysis therapy

**DOI:** 10.1111/cns.13759

**Published:** 2021-11-14

**Authors:** Qiwei Peng, Rentang Bi, Shengcai Chen, Jiefang Chen, Zhifang Li, Jianzhuang Li, Huijuan Jin, Bo Hu

**Affiliations:** ^1^ Department of Neurology Union Hospital Tongji Medical College Huazhong University of Science and Technology Wuhan China

**Keywords:** bilirubin subtype, ischemic stroke, metabolism, neurotoxicity, predictive value, thrombolysis

## Abstract

**Aims:**

To explore the association of total bilirubin (TBIL), direct bilirubin (DBIL), and indirect bilirubin (IBIL) levels with, as well as the incremental predictive value of different bilirubin subtypes for, poor outcomes in acute ischemic stroke patients after thrombolysis.

**Methods:**

We analyzed 588 individuals out of 718 AIS participants, and all patients were followed up at 3 months after thrombolysis. The primary outcome was 3‐month death and major disability (modified Rankin Scale (mRS) score of 3–6). The secondary outcomes were 3‐month mortality (mRS score of 6), moderate‐severe cerebral edema, and symptomatic intracranial hemorrhage (sICH), respectively.

**Results:**

Elevated DBIL pre‐thrombolysis was associated with an increased risk of primary outcome (OR 3.228; 95% CI 1.595–6.535; *p* for trend = 0.014) after fully adjustment. Elevated TBIL pre‐thrombolysis showed the similar results (OR 2.185; 95% CI 1.111–4.298; *p* for trend = 0.047), while IBIL pre‐thrombolysis was not significantly associated with primary outcome (OR 1.895; 95% CI 0.974–3.687; *p* for trend = 0.090). Multivariable‐adjusted spline regression model showed a positive linear dose‐response relationship between DBIL pre‐thrombolysis and risk of primary outcome (*p* for linearity = 0.004). Adding DBIL pre‐thrombolysis into conventional model had greater incremental predictive value for primary outcome, with net reclassification improvement (NRI) 95% CI = 0.275 (0.084–0.466) and integrated discrimination improvement (IDI) 95% CI = 0.011 (0.001–0.024). Increased DBIL post‐thrombolysis had an association with primary outcome (OR 2.416; 95%CI 1.184–4.930; *p* for trend = 0.039), and it also elevated the incremental predictive value for primary outcome, with NRI (95% CI) = 0.259 (0.066–0.453) and IDI (95% CI) = 0.025 (0.008–0.043).

**Conclusion:**

Increased DBIL pre‐thrombolysis had a stronger association with, as well as greater incremental predictive value for, poor outcomes than TBIL and IBIL did in AIS patients after thrombolysis, which should be understood in the context of retrospective design. The effect of DBIL on targeted populations should be investigated in further researches.

## Graphical abstract

Stroke is a systemic disease, with bilirubin metabolism enhanced in liver after stroke, exerting neurotoxicity in ischemia brain. Direct bilirubin (conjugated bilirubin) is superior in predicting the clinical outcomes in acute stroke patients receiving thrombolysis therapy than both total bilirubin and indirect bilirubin (unconjugated bilirubin).

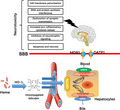

## 1 INTRODUCTION

Acute ischemic stroke (AIS) has a high disability and mortality rate, which brings a huge economic burden to the society and the family.^1^, ^2^ To date, thrombolytic therapy with recombinant tissue plasminogen activator (rt‐PA) and endovascular thrombectomy (EVT) are still the frontline treatment strategies for acute ischemic stroke within the time window.^3^ However, the overall effectiveness of these treatments has been reported to be limited, with only 30%–50% of patients achieving good long‐term outcomes.^4^, ^5^ Many patients receiving reperfusion therapy are at high risk of suffering from certain complications such as cerebral hemorrhage transformation and cerebral edema, who could not achieve good clinical outcome after discharge. To date, usefulness of a biomarker is limited to identify patients at high risk of getting worse clinical outcomes.^6^, ^7^ Therefore, alternative markers that have the potential to identify targeted patients pre‐thrombolysis to escalate preventive therapy are thus needed.

Bilirubin, a potent endogenous antioxidant, is produced in the heme catabolic pathway, with liver being the primary organ responsible for metabolism and excretion of bilirubin.^8^ Clinically, bilirubin levels are reported as total bilirubin (TBIL) and direct bilirubin (DBIL), and TBIL is the sum of DBIL and indirect bilirubin (IBIL).^9^ Previous studies reported that bilirubin exhibits both neurotoxic and neuroprotective effects after ischemic stroke, without reaching a consensus for the prognosis of ischemic stroke.^10^ However, these studies almost merely concentrated on one of the subtypes of bilirubin, without distinguishing the difference among them. To the best of our knowledge, studies on the predictive value of TBIL, DBIL, and IBIL for the clinical outcomes in patients diagnosed with AIS receiving intravenous thrombolysis are still lacking.

In this study, we analyzed the association of three subtypes of bilirubin pre‐thrombolysis with clinical outcomes as well as compared the performance of them as an indicator of worse outcomes among AIS patients receiving intravenous thrombolysis after ischemic stroke to elucidate that DBIL pre‐thrombolysis level has the potential to identify patients who are likely to be at increased risk of poor outcomes after intravenous thrombolysis to escalate preventive therapy.

## 2 METHODS

**FIGURE 1:**
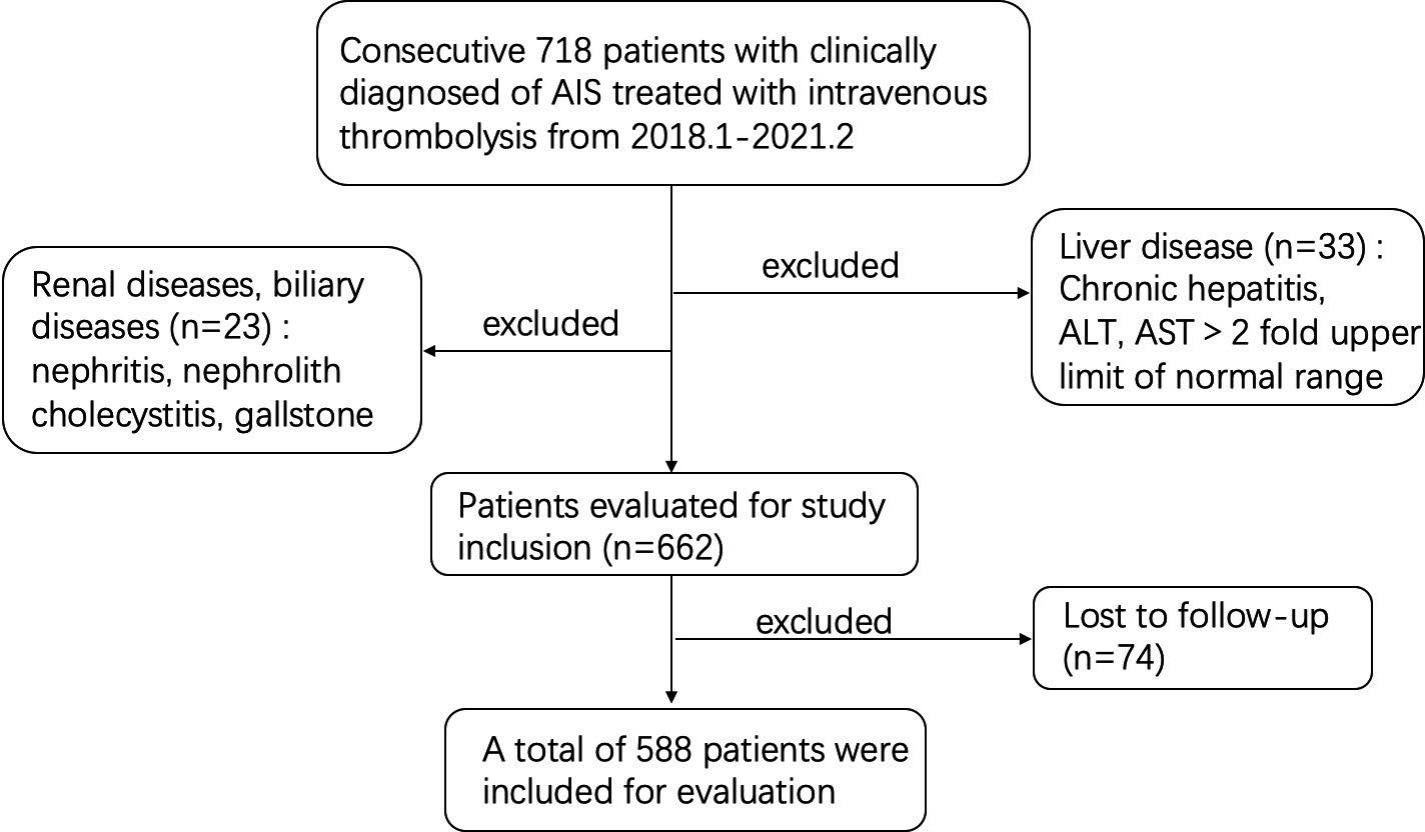
The flowchart of study population in this study

This retrospective study (Multicenter Clinical Trial of Revascularization Treatment for Acute Ischemic Stroke, TRAIS) was conducted among 718 AIS patients who received intravenous thrombolysis at 5 comprehensive stroke centers between January 2018 and February 2021 in China, including Wuhan Union Hospital, Wuhan Union Hospital West Campus, Central Hospital of Hefeng County, People's Hospital of Dongxihu District, and The Frist People's Hospital of Yichang City. We enrolled all AIS patients ≥18 years old who received intravenous thrombolysis therapy. Patients who had a diagnosis of (1) chronic hepatitis; (2) increased liver enzymes whose ALT or AST >twofold upper limit of normal range; (3) nephritis; (4) nephrolith; (5) cholecystitis; (6) gallstone as well as (7) who were lost to follow‐up were excluded (*n* = 130). All available hospitalization data, including medical history, clinical examination, laboratory examination, diagnostic examination, imaging examination and discharge diagnosis, were used for the diagnosis of the above diseases. A total of 588 participants were involved in final analysis (Figure 1). The ethics of the study conformed to the principles stated in the 1975 Declaration of Helsinki. The Ethics Committee of Union Hospital, Tongji Medical College, Huazhong University of Science and Technology approved all aspects of the study (ChiCTR2000033456). Written consent has been obtained from all participants in the study.

All the patients treated with thrombolytic treatment were in line with the written institutional guidelines. The time window for thrombolysis is extended and limited up to 9 h guided by perfusion imaging.^11^, ^12^ Intravenous rt‐PA injection (administered at a standard dose of 0.9 mg/kg body weight) was given according to the procedure recommended by the European Stroke Organization (ESO)^13^: 10% of the total dose being given as the first dose and the remaining dose being given within the next hour. Continuous monitoring and evaluation were conducted during thrombolysis procedure. After thrombolysis was completed, patients were transferred to the neurology intensive care unit (NICU) for intensive nursing.

Peripheral venous blood samples from patients were collected both pre‐thrombolysis upon admission and within 1–3 days post‐thrombolysis. Samples with hemolysis were discarded. The demographic characteristics, clinical features, and medical history of all enrolled patients were collected. Stroke severity was assessed both upon admission pre‐thrombolysis and post‐thrombolysis by trained neurologists using National Institutes of Health Stroke Scale (NIHSS). All routine laboratory examination results were obtained at emergency department (pre‐thrombolysis) and inpatient department (post‐thrombolysis). Hypertension is identified as one of the following conditions: blood pressure ≥140/90 mmHg or self‐reported physician‐diagnosed hypertension or current use of antihypertensive medication.^14^ According to Chinese guidelines on the prevention and treatment of hyperlipidemia, hyperlipidemia is considered as the abnormity of lipids in the blood (total cholesterol >6.22 mmol/L or triglyceride >2.26 mmol/L or low‐density lipoprotein cholesterol >4.14 mmol/L) or self‐reported history of physician diagnosis of hyperlipidemia.^15^ Patients with fasting glucose level >7.0 mmol/L or self‐reported physician‐diagnosed of diabetes or taking oral hypoglycemic drugs or insulin were defined as diabetes mellitus.^14^

Participants were followed up by modified Rankin Scale (mRS) score at 3 months by trained neurologists who were not aware of the treatment allocation. The primary outcome was defined as 3‐month death and major disability (mRS score of 3–6), and secondary outcomes were defined as (1) 3‐month mortality (mRS score of 6); (2) moderate‐severe cerebral edema, defined as those with swelling area greater than 1/3 of the hemisphere based on cerebral CT or magnetic resonance imaging (MRI) within 1–3 days post‐thrombolysis, according to Safe Implementation of Thrombolysis in Stroke‐Monitoring Study (SITS‐MOST) criteria^16^; (3) symptomatic intracranial hemorrhage (sICH), defined as any hemorrhagic transformation temporarily associated with deterioration of neurological symptoms using the National Institute of Neurological Disorders and Stroke (NINDS) criteria.^4^

### 2.1 Statistical analysis

To analyze the association of three subtypes of bilirubin with clinical outcomes, the participants were divided into 4 groups according to quartiles of TBIL, IBIL and DBIL, respectively. Kolmogorov–Smirnov (K–S) test of normality was used to assess data distribution. Continuous variables with normal distributions were represented as mean ± standard difference (SD), while other variables were represented as median (interquartile range). Categorical variables were represented as numbers (percentages). The quartile differences of the baseline characteristics of each bilirubin subtype were tested with chi‐square tests for categorical characteristics and analysis of variance (ANOVA) for continuous characteristics. Binary logistic regression models were used to estimate the relationship of three subtypes of bilirubin with primary and secondary outcomes. Odds ratios (ORs) and 95% confidence intervals (CIs) of primary and secondary clinical outcomes for higher quartiles compared with the lowest quartile and for each SD increase of log‐transformed of three subtypes bilirubin were calculated. We constructed two models with progressive adjustment: model 1 was adjusted for age, sex, onset‐time to treatment (OTT), admission glucose level, admission ALT, admission AST, cigarette smoking, alcohol drinking, history of stroke, cerebral hemorrhage, hypertension, diabetes mellitus and hyperlipidemia; model 2 was additionally adjusted for admission NIHSS score. We tested the linear trends across the quartiles of three subtypes of bilirubin by including the quartiles in the models as continuous variable. Restricted cubic spline (RCS) model with knots at the 5th, 35th, 65th, and 95th percentiles^17^ was used to characterize the shape of the association of bilirubin level with primary outcome. Receiver operating characteristic (ROC) curve which is equivalent to the C‐statistic was constructed to estimate the discriminative power of three subtypes bilirubin for primary and secondary outcomes as well as compare the discriminative power of DBIL both pre‐ and post‐thrombolysis for primary outcome. The predictive power of each bilirubin pre‐thrombolysis when added to conventional model (CM) was assessed by net reclassification improvement (NRI) and integrative discriminative improvement (IDI).^18^ Additionally, subgroup analysis was performed to assess the potential modified effect of 12 interesting factors on the association between DBIL pre‐thrombolysis and primary outcome. A two‐sided *p* value <0.05 was considered to be statistically significant. Statistical analyses were carried out using R software (version 4.0.3) and MedCalc 15.2.0 (MedCalc Software, Mariakerke, Belgium).

## 3 RESULTS

### 3.1 Characteristics of study population

Overall, the mean age of participants at baseline was 64.7 years, and 66.4% of them were men. The median (interquartile range) overall were 3.6 (2.7–5.1) μmol/L for DBIL, 10.4 (7.8–14.2) μmol/L for TBIL, 6.8 (4.8–9.5) μmol/L for IBIL, respectively. Baseline characteristics of participants by DBIL level quartiles are shown in Table 1. Participants with higher DBIL were more likely to be older; to have higher admission NIHSS score; to have longer OTT; to have higher liver enzyme level of AST; and to have higher prevalence of 3‐month mRS score of 3–6, 3‐month mRS score of 6, moderate‐severe cerebral edema as well as sICH; In contrast, the prevalence of hyperlipidemia decreased as DBIL level increased. And the similar characteristics of the study population by TBIL and IBIL levels are shown in Tables S1 and S2.

**TABLE 1:** Baseline characteristics of participants across quartiles of serum direct bilirubin levels

Characteristics	Total	Serum direct bilirubin, μmol/L				*p* Value for Trend
		Q1 (<2.7)	Q2 (2.7–3.6)	Q3 (3.6–5.1)	Q4 (≥5.1)	
Patients, n	585	142	139	157	147	
Age (year)	64.9 ± 12.2	62.0 ± 11.1	64.9 ± 11.8	66.3 ± 12.5	65.9 ± 12.9	0.004**
male, *n* (%)	389 (66.5)	95 (66.9)	96 (69.1)	108 (68.8)	90 (61.2)	0.722
History of ischemic stroke	82 (14.0)	25 (17.6)	20 (14.4)	19 (12.1)	18 (12.2)	0.524
History of intracerebral hemorrhage	13 (2.2)	6 (4.2)	5 (3.6)	0	2 (1.4)	0.238
History of hypertension	378 (64.6)	92 (64.8)	80 (57.6)	109 (69.4)	97 (65.9)	0.922
History of hyperlipidemia	123 (21.0)	34 (23.9)	27 (19.4)	25 (15.9)	21 (14.3)	0.012*
History of diabetes mellitus	164 (28.0)	35 (24.6)	36 (25.9)	53 (33.8)	40 (27.2)	0.625
Current cigarette smoking	202 (34.5)	44 (31.0)	44 (31.7)	64 (40.8)	50 (34)	0.376
Current alcohol drinking	118 (20.2)	24 (16.9)	25 (18.0)	34 (21.7)	35 (23.8)	0.16
Admission NIHSS score	4.0 (2.0–8.0)	4.0 (2.0–7.0)	3.0 (1.0–6.0)	4.0 (2.0–7.5)	4.0 (2.0–11.0)	0.04*
OTT, min	189.0 (136.5–250)	169.5 (117.5–215.0)	189.0 (140.0–240.0)	216.8 (140.1–259.5)	210.0 (149.0–260.0)	<0.001***
Admission glucose, mmol/L	6.9 (5.4–8.8)	6.8 (5.5–8.8)	6.5 (5.3–8.2)	7.0 (5.5–9.10)	7.1 (5.4–9.3)	0.942
Admission ALT, μmol/L	17.0 (12.0–24.0)	17.5 (13.0–23.0)	16.0 (12.0–22.0)	16.0 (12.0–24.0)	18.0 (12.0–27.0)	0.537
Admission AST, μmol/L	20.0 (16.0–25.0)	20.0 (16.0–24.0)	19.0 (16.0–22.0)	19.0 (16.0–25.0)	21.0 (16.75–28.0)	0.044*
3‐month death and major disability, *n* (%)	150 (25.6)	24 (16.9)	34 (24.5)	38 (24.2)	54 (36.7)	<0.001***
3‐month mortality, *n* (%)	47 (8.0)	4 (2.8)	10 (7.2)	14 (8.9)	19 (12.9)	0.008**
Moderate‐severe cerebral edema, *n* (%)	64 (10.9)	12 (8.5)	14 (10.1)	11 (7.0)	27 (18.4)	0.001**
sICH, *n* (%)	35 (5.9)	6 (4.2)	7 (5.0)	5 (3.2)	17 (11.6)	0.009**

Abbreviations: ALT, Alanine aminotransferase; AST, Aspartate aminotransferase; NIHSS, National Institutes of Health Stroke Scale; OTT, onset‐to‐treatment time; sICH, symptomatic intracranial hemorrhage.

* *p* < 0.05.

** *p* < 0.01.

*** *p* < 0.001.

### 3.2 *Association of different bilirubin subtypes pre*‐*thrombolysis with primary outcome*

**FIGURE 2:**
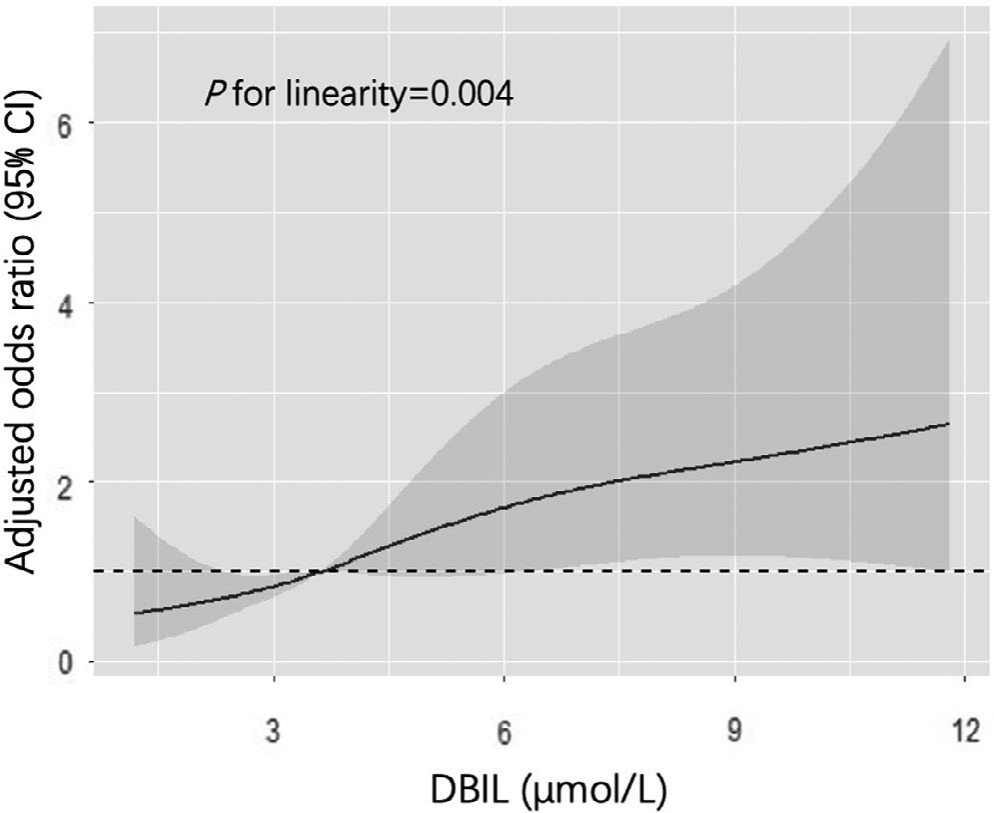
Fully adjusted odds ratios (ORs) of primary outcome according to DBIL pre‐thrombolysis. OR and 95% CI derived from restricted cubic spline regression. OR adjusted for the same variables as model 2 in Table 2

As shown in Table 2, compared with first quartile of DBIL, the fully adjusted OR from the second to the fourth quartile in model 2 were 2.225 (1.072–4.617), 2.197 (1.068–4.520), and 3.228 (1.595–6.535), respectively (*p* for trend = 0.014). The fully adjusted OR from the second to the fourth quartile of TBIL in model 2 were 0.973 (0.481–1.972), 1.244 (0.624–2.478), and 2.185 (1.111–4.298), respectively (*p* for trend = 0.047), compared with the first quartile. As for IBIL, it was not significantly associated with the primary outcome, showing the fully adjusted OR from the second to the fourth quartile in model 2 were 1.238 (0.631–2.430), 0.875 (0.433–1.771), and 1.895 (0.974–3.687), respectively (*p* for trend = 0.090), compared with the first quartile. It was worth noting that each SD increase of log‐transformed DBIL had higher OR associated with primary outcome in model 2 (OR 1.457, 95% CI 1.163–1.824), compared with TBIL (OR 1.344, 95% CI 1.083–1.666), indicating that DBIL had a stronger association with primary outcome after fully adjustment than TBIL or IBIL. Simultaneously, the dose–response relationship between DBIL and primary outcome was further demonstrated with RCS (*p* for linearity = 0.004; Figure 2).

**TABLE 2:** Odds ratios and 95% CI of primary outcome for quartiles of each serum bilirubin pre‐thrombolysis

Bilirubin types	No. of cases, n (%)	Odds ratios (95% CI)	
		Model 1	Model 2
Total bilirubin	151 (25.7)	—	—
Quartile 1	28 (19.6)	1.00 (Ref.)	1.00 (Ref.)
Quartile 2	34 (22.7)	1.150 (0.645–2.049)	0.973 (0.481–1.972)
Quartile 3	37 (25.2)	1.351 (0.764–2.390)	1.244 (0.624–2.478)
Quartile 4	52 (35.1)	2.198 (1.263–3.824)	2.185 (1.111–4.298)
*p* for trend	—	0.023*	0.047*
Each SD increase of log‐total bilirubin	—	1.334 (1.109–1.606)	1.344 (1.083–1.666)
Indirect bilirubin	151 (25.7)	—	—
Quartile 1	32 (21.8)	1.00 (Ref.)	1.00 (Ref.)
Quartile 2	41 (27.9)	1.352 (0.779–2.344)	1.238 (0.631–2.430)
Quartile 3	28 (19.2)	0.874 (0.489–1.561)	0.875 (0.433–1.771)
Quartile 4	50 (33.8)	1.688 (0.986–2.890)	1.895 (0.974–3.687)
*p* for trend	—	0.072	0.090
Each SD increase of log‐indirect bilirubin	—	1.223 (1.020–1.468)	1.263 (1.022–1.560)
Direct bilirubin	150 (25.6)	—	—
Quartile 1	23 (16.2)	1.00 (Ref.)	1.00 (Ref.)
Quartile 2	33 (23.7)	1.702 (0.927–3.127)	2.225 (1.072–4.617)
Quartile 3	40 (25.5)	1.934 (1.062–3.523)	2.197 (1.068–4.520)
Quartile 4	54 (36.7)	3.363 (1.869–6.050)	3.228 (1.595–6.535)
*p* for trend	—	0.001**	0.014*
Each SD increase of log‐direct bilirubin	—	1.501 (1.245–1.810)	1.457 (1.163–1.824)

Note

Model 1: Adjusted for age, sex, onset‐time to treatment, admission glucose, admission ALT, admission AST, current smoking, alcohol drinking, history of stroke, cerebral hemorrhage, hypertension, diabetes mellitus, and hyperlipidemia.

Model 2: Model 1+ admission NIHSS score.

* *p* < 0.05.

** *p* <.01

### 3.3 *Association of different bilirubin subtypes pre*‐*thrombolysis with secondary outcomes*

As shown in Table 3, compared with first quartile of DBIL, the fully adjusted OR of 3‐month mortality from the second to the fourth quartile in model 2 were 3.002 (0.748–11.502), 4.499 (1.231–16.443), and 5.872 (1.671–20.640), respectively (*p* for trend = 0.041). The fully adjusted OR of 3‐month mortality from the second to the fourth quartiles of TBIL in model 2 were 0.523 (0.175–1.563), 0.736 (0.258–2.102), and 1.927 (0.758–4.899), respectively (*p* for trend = 0.043), compared with the first quartile. As for IBIL, it was not significantly associated with the 3‐month mortality, showing the fully adjusted OR from the second to the fourth quartile in model 2 were 0.450 (0.152–1.329), 0.902 (0.338–2.408) and 1.524 (0.617–3.763), respectively (*p* for trend = 0.139), compared with the first quartile. It was also worth noting that each SD increase of log‐transformed DBIL had higher OR associated with 3‐month mortality in model 2 (OR 1.557, 95% CI 1.090–2.224), compared with TBIL (OR 1.246, 95% CI 0.919–1.689), indicating that DBIL had a stronger association with 3‐month mortality than TBIL or IBIL. Similar results were observed for association of DBIL, TBIL and IBIL with moderate‐severe cerebral edema, respectively (shown in Table S3). In addition, none of DBIL, TBIL, and IBIL was in association with sICH (shown in Table S4).

**TABLE 3:** Odds ratios and 95% CI of 3‐month mortality for quartiles of each serum bilirubin pre‐thrombolysis

Bilirubin types	No. of cases, n (%)	Odds ratios (95% CI)	
		Model 1	Model 2
Total bilirubin	47 (7.9)	—	—
Quartile 1	10 (7.0)	1.00 (Ref.)	1.00 (Ref.)
Quartile 2	8 (5.3)	0.645 (0.240–1.735)	0.523 (0.175–1.563)
Quartile 3	9 (6.1)	0.782 (0.301–2.036)	0.736 (0.258–2.102)
Quartile 4	20 (13.5)	1.877 (0.820–4.296)	1.927 (0.758–4.899)
*p* for trend	—	0.057	0.043*
Each SD increase of log‐total bilirubin	—	1.211 (0.918–1.597)	1.246 (0.919–1.689)
Indirect bilirubin	47 (7.9)	—	—
Quartile 1	12 (8.2)	1.00 (Ref.)	1.00 (Ref.)
Quartile 2	7 (4.8)	0.536 (0.201–1.430)	0.450 (0.152–1.329)
Quartile 3	10 (6.8)	0.763 (0.311–1870)	0.902 (0.338–2.408)
Quartile 4	18 (12.2)	1.331 (0.597–2.968)	1.524 (0.617–3.763)
*p* for trend	—	0.237	0.139
Each SD increase of log‐indirect bilirubin	—	1.161 (0.883–1.526)	1.217 (0.906–1.635)
Direct bilirubin	47 (8.0)	—	—
Quartile 1	4 (2.8)	1.00 (Ref.)	1.00 (Ref.)
Quartile 2	9 (6.5)	2.403 (0.707–8.168)	3.002 (0.784–11.502)
Quartile 3	15 (9.6)	4.126 (1.277–13.330)	4.499 (1.231–16.443)
Quartile 4	19 (12.9)	5.651 (1.797–17.769)	5.872 (1.671–20.640)
*p* for trend	—	0.017*	0.041*
Each SD increase of log‐direct bilirubin	—	1.638 (1.187–2.261)	1.557 (1.090–2.224)

Note

Model 1: Adjusted for age, sex, onset‐time to treatment, admission glucose, admission ALT, admission AST, current smoking, alcohol drinking, history of stroke, cerebral hemorrhage, hypertension, diabetes mellitus, and hyperlipidemia.

Model 2: Model 1+ admission NIHSS score.

* *p* < 0.05.

### 3.4 *Performance of different bilirubin subtypes pre*‐*thrombolysis as a biomarker for different clinical outcome*

**FIGURE 3:**
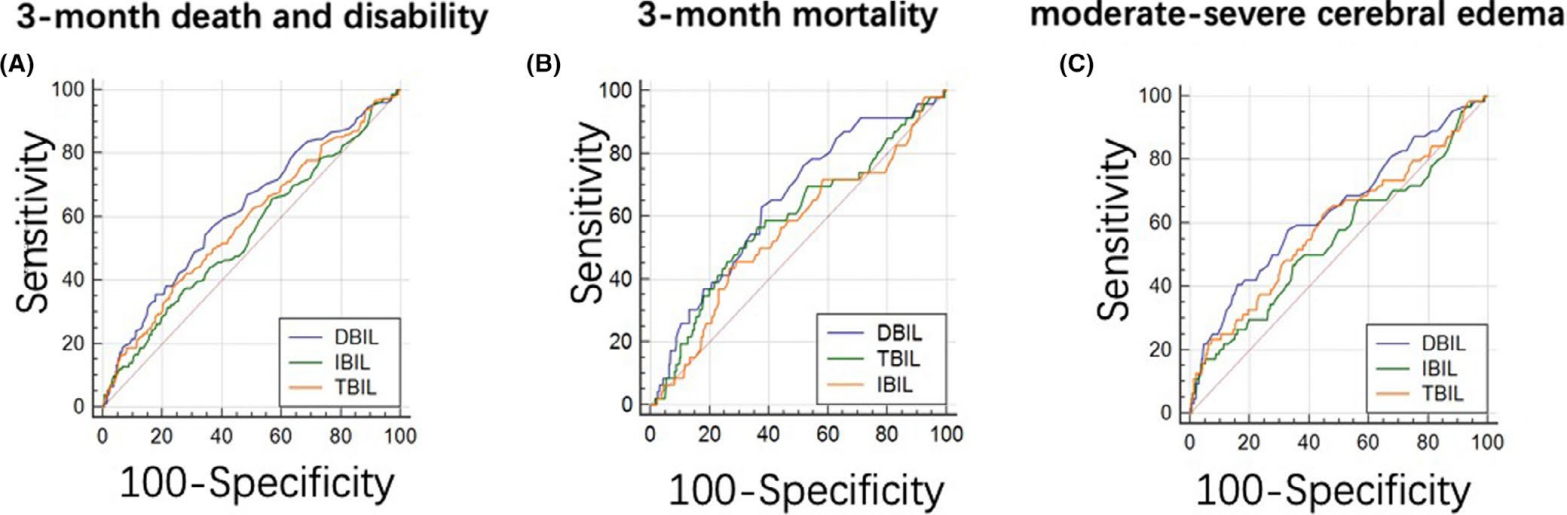
ROC analysis of three subtypes bilirubin for 3‐month death and disability (A), 3‐month mortality (B), and moderate‐severe cerebral edema (C)

In the ROC analysis shown in Figure 3 and Table S5, DBIL, TBIL and IBIL evaluated separately showed poor‐moderate discriminative powers for primary (C‐statistic 0.622, 95% CI 0.569–0.675 for DBIL, C‐statistic 0.585, 95% CI 0.530–0.639 for TBIL and C‐statistic 0.548, 95% CI 0.493–0.603 for IBIL) and secondary outcomes including 3‐month mortality (C‐statistic 0.648, 95% CI 0.567–0.729 for DBIL, C‐statistic 0.591, 95% CI 0.501–0.681 for TBIL and C‐statistic 0.522, 95% CI 0.462–0.642 for IBIL) and moderate‐severe cerebral edema (C‐statistic 0.640, 95% CI 0.562–0.718 for DBIL, C‐statistic 0.593, 95% CI 0.512–0.674 for TBIL and C‐statistic 0.549, 95% CI 0.467–0.631 for IBIL), with DBIL possessing the highest discriminative power over TBIL or IBIL (*p* < 0.05).

To further explore the incremental predictive value of different bilirubin subtypes for primary and secondary outcomes, we evaluated the effect of adding them to multiparameter CM which is the same as the risk factors in fully adjusted model 2, respectively. As shown in Table 4, compared with both TBIL and IBIL, the addition of DBIL to the CM allowed a significant incremental prediction of risk for 3‐month death and major disability with NRI (95% CI) = 0.275 (0.084–0.466) and IDI (95% CI) = 0.011 (0.001–0.024), which was superior to that of TBIL with NRI (95% CI) = 0.188 (−0.002–0.377) and IDI (95% CI) = 0.007 (−0.003–0.016), and IBIL with NRI (95% CI) = 0.087 (−0.102–0.276) and IDI (95% CI) = 0.004 (−0.004–0.012). The C‐statistic for model including DBIL tended to be higher than for the CM alone, with C‐statistic (95% CI) for the CM = 0.825 (0.789–0.857), C‐statistic (95% CI) including DBIL = 0.835 (0.800–0.867), which was superior to that of TBIL with C‐statistic (95% CI) = 0.832 (0.797–0.864) and IBIL with C‐statistic (95% CI) = 0.830 (0.794–0.862). Similar results could be observed by adding three different bilirubin subtypes to CM for 3‐month mortality and moderate‐severe cerebral edema (Tables S6 and S7).

**TABLE 4:** Incremental predictive value of different bilirubin subtypes for primary outcome

	Discrimination		Reclassification			
	C‐statistic (95% CI)	*p* value	NRI (95% CI)	*p* value	IDI (95% CI)	*p* value
Conventional model (CM)	0.825 (0.789–0.857)	—	1.00 (Ref.)	—	1.00 (Ref.)	—
CM + TBIL	0.832 (0.797–0.864)	0.241	0.188 (−0.002–0.377)	0.052	0.007 (−0.003–0.016)	0.178
CM + IBIL	0.830 (0.794–0.862)	0.377	0.087 (−0.102–0.276)	0.367	0.004 (−0.004–0.012)	0.303
CM + DBIL	0.835 (0.800–0.867)	0.141	0.275 (0.084–0.466)	0.005**	0.011 (0.001–0.024)	0.037*

Note

CM: age, sex, onset‐time to treatment, admission NIHSS score, admission glucose, admission ALT, admission AST, current smoking, alcohol drinking, history of stroke, cerebral hemorrhage, hypertension, diabetes mellitus, and hyperlipidemia.

* *p* < 0.05.

** *p* < 0.01.

### 3.5 Subgroup analysis for association between DBIL and primary outcome

**FIGURE 4:**
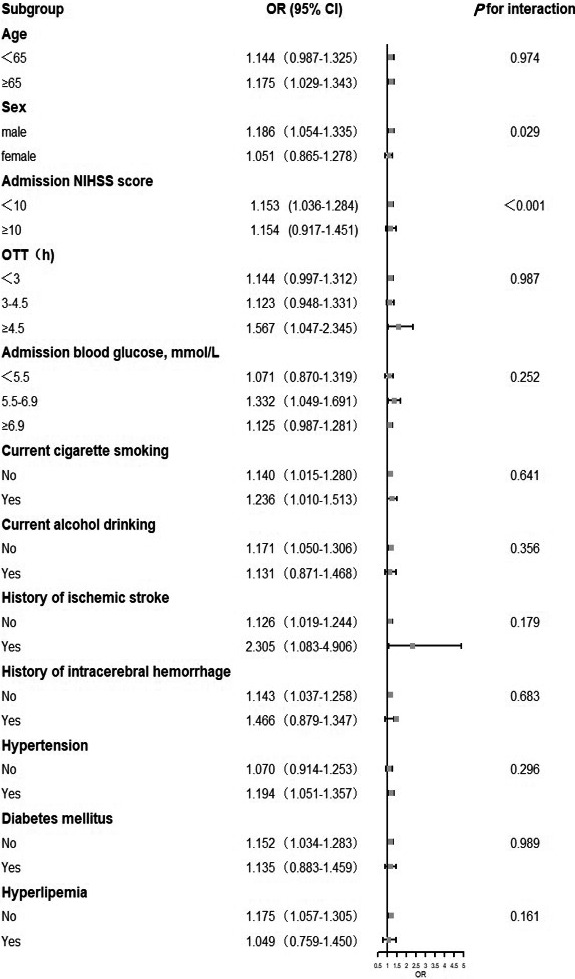
Subgroup analyses of the association between DBIL pre‐thrombolysis and primary outcome. Interactions between DBIL and interesting factors on the primary outcome were tested by the likelihood ratio test with adjustment for the same variables in model 2. Odds ratio and 95% CIs were shown by forest plot

We performed stratified analysis for the dose–response association of DBIL pre‐thrombolysis with primary clinical outcome according to prespecified factors. The significant interaction was only found between DBIL and admission NIHSS score ≤10 (*p* for interaction <0.001, Figure 4).

### 3.6 *Association and Performance of DBIL Post*‐*thrombolysis for Primary Outcome*

Firstly, we compared the concentration of DBIL pre‐ and post‐thrombolysis, showing that concentration of DBIL post‐thrombolysis was higher than that of pre‐thrombolysis (*p* < 0.001, Figure S1). Next, we investigated the association of DBIL post‐thrombolysis with primary outcome. Compared with first quartile of DBIL, the fully adjusted OR from the second to the fourth quartile in model 2 were 1.935 (0.921–4.066), 1.547 (0.744–3.217) and 2.416 (1.184–4.930), respectively (*p* for trend = 0.039). And each SD increase of log‐transformed DBIL was associated with 56.2% increased risk of primary outcome in model 2 (Table S8). Furthermore, we explored the incremental predictive value of DBIL for primary outcome as well. As shown in Table S9, the addition of DBIL to the CM allowed a significant incremental prediction of risk with NRI (95% CI) = 0.259 (0.066–0.453) and IDI (95% CI) = 0.025 (0.008–0.043). However, the overall discriminative power comparison of DBIL pre‐ and post‐thrombolysis for primary outcome applied by ROC curves showed that the discriminative power difference was not significant (*p* = 0.908, Figure S2).

## 4 DISCUSSION

Previously, little is known about the effect of individual bilirubin subtype on clinical outcomes of ischemic stroke patients who received thrombolysis therapy. In the present study, we observed a stronger association with and superior predictive value of DBIL pre‐thrombolysis for 3‐month death and major disability, 3‐month mortality as well as moderate‐severe cerebral edema but not sICH compared with TBIL or IBIL. To the best of our knowledge, this is the first retrospective study to investigate the relationship between different subtypes of bilirubin and poor clinical outcomes in the targeted patients.

The association of TBIL and outcomes in stroke patients without reperfusion treatment has been reported in the past, while the results were inconsistent. For instance, Oda et al. showed that lower quartiles of TBIL were associated with greater prevalence of stroke,^19^ whereas Kurzepa et al. suggested that higher TBIL to be a poor clinical outcome factor for ischemic stroke.^20^ As for IBIL, to our knowledge, only one result pointed out that higher IBIL was in association with mortality in AIS patients without reperfusion treatment.^21^ To date, DBIL has been investigated as a biomarker in many epidemiological studies, reported that higher DBIL was in a positive association with coronary heart disease,^14^ stroke severity,^22^ type 2 diabetes mellitus,^23^ and diabetic microvascular complications^24^ compared with TBIL and IBIL. All the above studies indicated that higher DBIL tended to be a risk factor rather than a potential antioxidant in oxidative stress‐mediated disease. In our study, we validated DBIL to be more suitable and superior than TBIL and IBIL for prediction of poor outcomes after thrombolysis. In contrast, a recent study reported that TBIL and IBIL but not DBIL were both independent risk factors for sICH in AIS patients receiving EVT therapy,^25^ and the contrast result may be attributed to adjustment of different covariates, variations in study sampling as well as different diagnosis criteria for sICH. Particularly, in our subgroup analysis, no significant interaction between DBIL pre‐thrombolysis and these subgroup characteristics except for admission NIHSS score was found, showing a stronger association in individuals with admission NIHSS score ≤10 (mild‐moderate stroke), while the underlying mechanism for this observation was unclear which needs further exploration. What's more, DBIL post‐thrombolysis was in association with, as well as provided excellent predictive value for, 3‐month death and major disability. While the difference of discriminative accuracy for DBIL between pre‐ and post‐thrombolysis was not significant, which meant that both DBIL pre‐ and post‐thrombolysis levels were equivalent in predicting the primary outcome. Importantly, DBIL measured pre‐thrombolysis would be much more clinically relevant, which may aid in predicting the risk of 3‐month death and major disability. And DBIL post‐thrombolysis may help clinicians decide which patients should be monitored more closely after thrombolysis.

As a systemic disease, ischemic stroke causes damage to other remote organs in the body, altering their signaling and metabolisms, including the liver.^26^ A large accumulation of data has demonstrated that leukocytes infiltration could also occur inside liver after brain ischemia reperfusion, accelerating the body to discharge more inflammatory factors, producing more reactive oxygens species (ROS) thus exacerbating the redox imbalance, intensifying endoplasmic reticulum (ER) stress and increasing the expression of heme oxygenase‐1 (HO‐1) in liver, which ultimately results in more bilirubin entering into bloodstream.^27^, ^28^, ^29^, ^30^ HO‐1, a key rate‐limiting enzyme of bilirubin production abundantly expressed in the spleen and liver,^29^ catalyzes the degradation of heme into three end‐products, namely carbon monoxide (CO), ferrous ion and biliverdin, among which biliverdin is rapidly reduced to bilirubin by biliverdin reductase (Figure S3), maintaining the bilirubin content in a dynamic balance in body under normal condition.^31^, ^32^ About 96% of bilirubin in normal plasma flows in an unconjugated form (i.e. indirect bilirubin) and is bound tightly to albumin to be transferred to the liver for the production of the conjugated form (i.e. direct bilirubin).^9^ Although the exact mechanism of bilirubin uptake into hepatocyte is not that clear, it appears that circulating bilirubin dissociates from albumin before entering hepatocytes by organic anion transporters (OATP) family, particularly OATP1B1 and OATP1B3, which are present in the lipid bilayer of liver cell membranes.^33^ Once in the hepatocytes of the liver, glucuronic acid is added to the unconjugated bilirubin by UDP‐glucuronosyltransferase (UGT1A1), forming the conjugated bilirubin.^8^ On the one hand, the newly conjugated bilirubin, secreted by the multidrug‐resistant protein MRP2 (ABCC2), enters into the bile through canalicular membrane. On the other hand, MRP3 (ABCC3) deposited the conjugated bilirubin back into the blood.^34^ The uptake of bilirubin into brain at the blood–brain barrier (BBB) may occur via one of organic anion transporters OATP1, and the efflux of bilirubin from the brain may be mediated by ATP‐binding cassette subfamily B member 1 (ABCB1; also referred to as MDR1 P‐glycoprotein),^35^ and the expression of OATP1 and MDR1 at the BBB was reported to be upregulated after brain ischemia (Figure S4).^35^, ^36^, ^37^

Previously, most studies have investigated the mechanisms linking elevated bilirubin and neurotoxicity although without fully understood. It has been shown that higher levels of bilirubin could directly interact with neuronal cell membrane phospholipids, interfere with DNA and protein synthesis as well as alter the intracellular pH.^38^, ^39^, ^40^, ^41^ Additionally, bilirubin could induce synaptic dysfunction resulting in reduced synaptic activation,^42^ stimulate the release of pro‐inflammatory cytokines such as interleukin‐1β (IL‐1β), IL‐6, and tumor necrosis factor‐α (TNF‐α) via activating the member of mitogen‐activated protein kinase (MAPK) family like p38 and c‐Jun N‐terminal kinase 1/2 (JNK1/2),^43^, ^44^ and inhibit brain derived neurotrophic factor‐induced activation of pro‐survival signaling, such as Akt‐protein kinase B system.^45^ Furthermore, not only caspase‐3 but also caspase‐8 and caspase‐9 could be activated by bilirubin, leading to neuronal apoptosis and necrosis,^46^, ^47^ and N‐methyl‐D‐aspartate (NMDA)‐induced glutamate release and excitotoxic cell death were observed to be increased at higher concentration of bilirubin.^48^, ^49^ Bilirubin can also directly induce glial death,^50^, ^51^, ^52^, ^53^ and it seems that neurons are more susceptible to bilirubin toxicity than astrocytes.^54^ Actually, the integrity of BBB is broken after stroke, resulting in the increased entry of bilirubin from peripheral circulation into brain to exert neurotoxic effect. Additionally, as all types of cell could maintain certain concentration of bilirubin both at physiological and pathological condition,^55^ it is therefore reasonable to speculate that brain cells could release intracellular bilirubin into bloodstream after brain ischemia reperfusion because of cell damage, together with peripheral‐derived bilirubin, resulting in hyperbilirubinemia. In a word, hyperbilirubinemia after brain ischemia reperfusion probably acts as a cell injury marker in blood and may produce neurotoxicity that exacerbates brain edema and reperfusion injuries, leading to poor prognosis in targeted patients, which requires further research.

However, the exact mechanisms linking DBIL and poor clinical outcomes of thrombolysis are still unclear, and some possible explanations could be proposed. DBIL is more soluble in serum than lipophilic IBIL after conjugation and bound weakly to albumin, thus making DBIL an active form of bilirubin more readily available than IBIL.^56^, ^57^ Meanwhile, as a systemic disease, elevated level of DBIL may indicate the injury of hepatocytes, whereas TBIL is within the normal range after stroke^58^; therefore, the positive association of DBIL levels with poor clinical outcomes might reflect the relationship between hepatic dysfunction and poor clinical outcomes. Future studies are thus required to elucidate the specific differences of different bilirubin subtypes with respect to their molecular mechanisms of action.

There were several limitations in our study. Firstly, the current study is a retrospective study with selection bias conducted in middle‐aged and elderly Chinese population, and further research on populations of different ethnic and age is needed to confirm our findings. Secondly, our sample size is not large enough which may have an influence on the results. Extensive large sample studies are needed to explore the underlying mechanisms of bilirubin and poor clinical outcomes in future. Thirdly, HO‐1 plays an important role in the effects of bilirubin generation, but we did not measure the levels of HO‐1 as well as markers of inflammation and oxidative stress in the population. Last but not the least, three subtypes of bilirubin were only assessed at two time points and the follow‐up period of this study was relatively short.

## 5 CONCLUSIONS

We found that increased DBIL pre‐thrombolysis had a stronger association with, as well as significantly improved the risk prediction of, poor clinical outcomes of 3‐month death and major disability, 3‐month mortality, and moderate‐severe cerebral edema than TBIL and IBIL in AIS patients receiving thrombolysis therapy, where the results of this study should be understood in the context of retrospective design. The effect of DBIL on poor outcomes should be noted, and the association between different bilirubin subtypes and clinical outcomes are warranted to be investigated in additional follow‐up studies.

## CONFLICT OF INTEREST

The authors declare no conflicts of interest.

## Supporting information

Fig S1

Fig S2

Fig S3

Fig S4

Table S1

Table S2

Table S3

Table S4

Table S5

Table S6

Table S7

Table S8

Table S9

## Data Availability

The data that support the findings of this study are available from the corresponding author upon reasonable request.
