# Supplementary figures and images for: Predictive value of different bilirubin subtypes for clinical outcomes in patients with acute ischemic stroke receiving thrombolysis therapy
Source: CNS Neurosci Ther. 2021 Nov 14;28(2):226–36. doi: 10.1111/cns.13759 (PMC8739039; doi:10.1111/cns.13759)

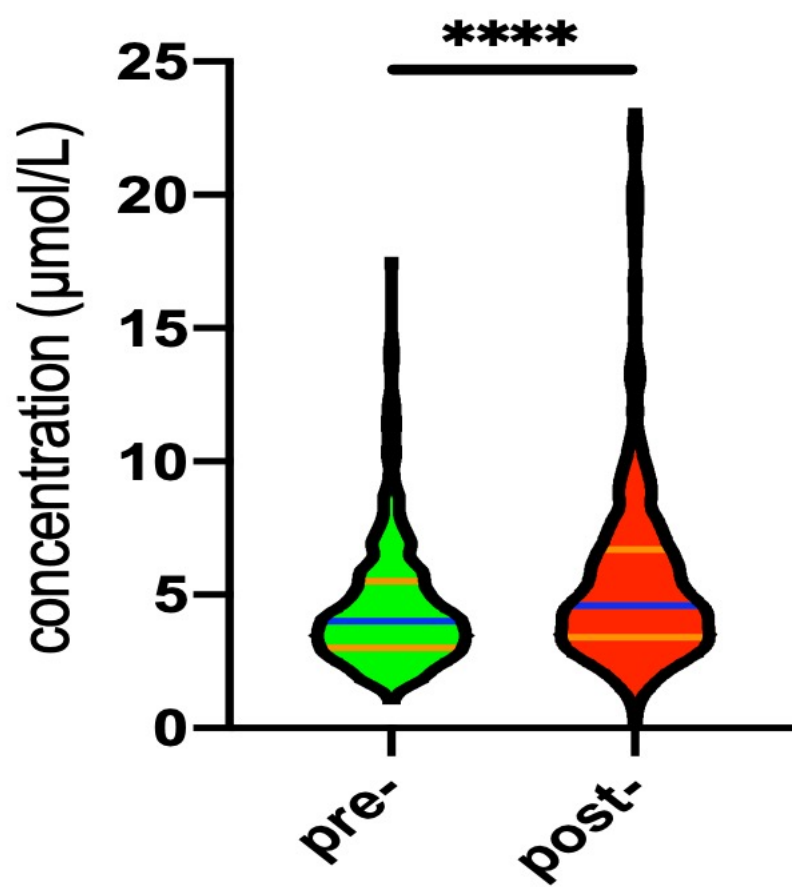

Supplement: Supplementary file 1 — Fig S1 [file CNS-28-226-s010.pdf]

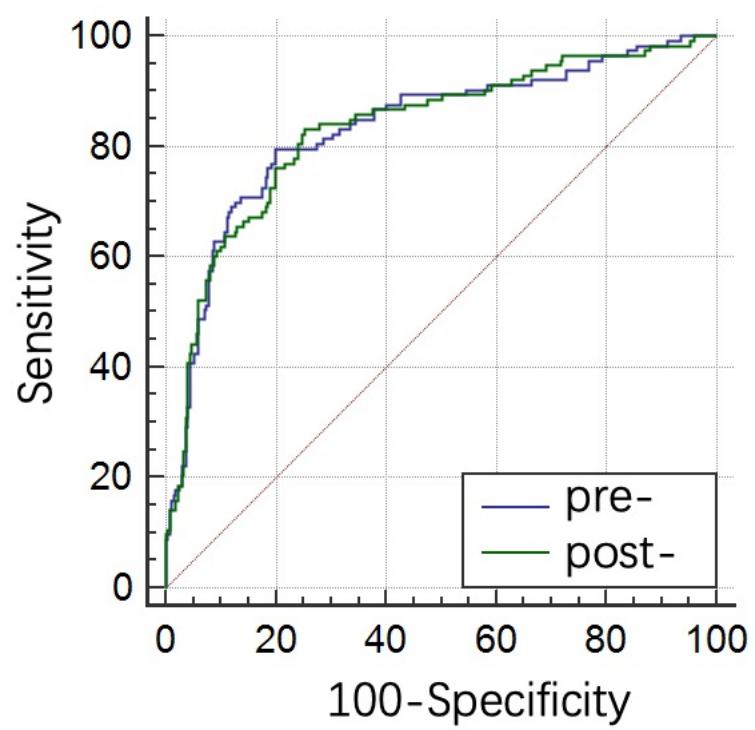

Supplement: Supplementary file 2 — Fig S2 [file CNS-28-226-s001.pdf]

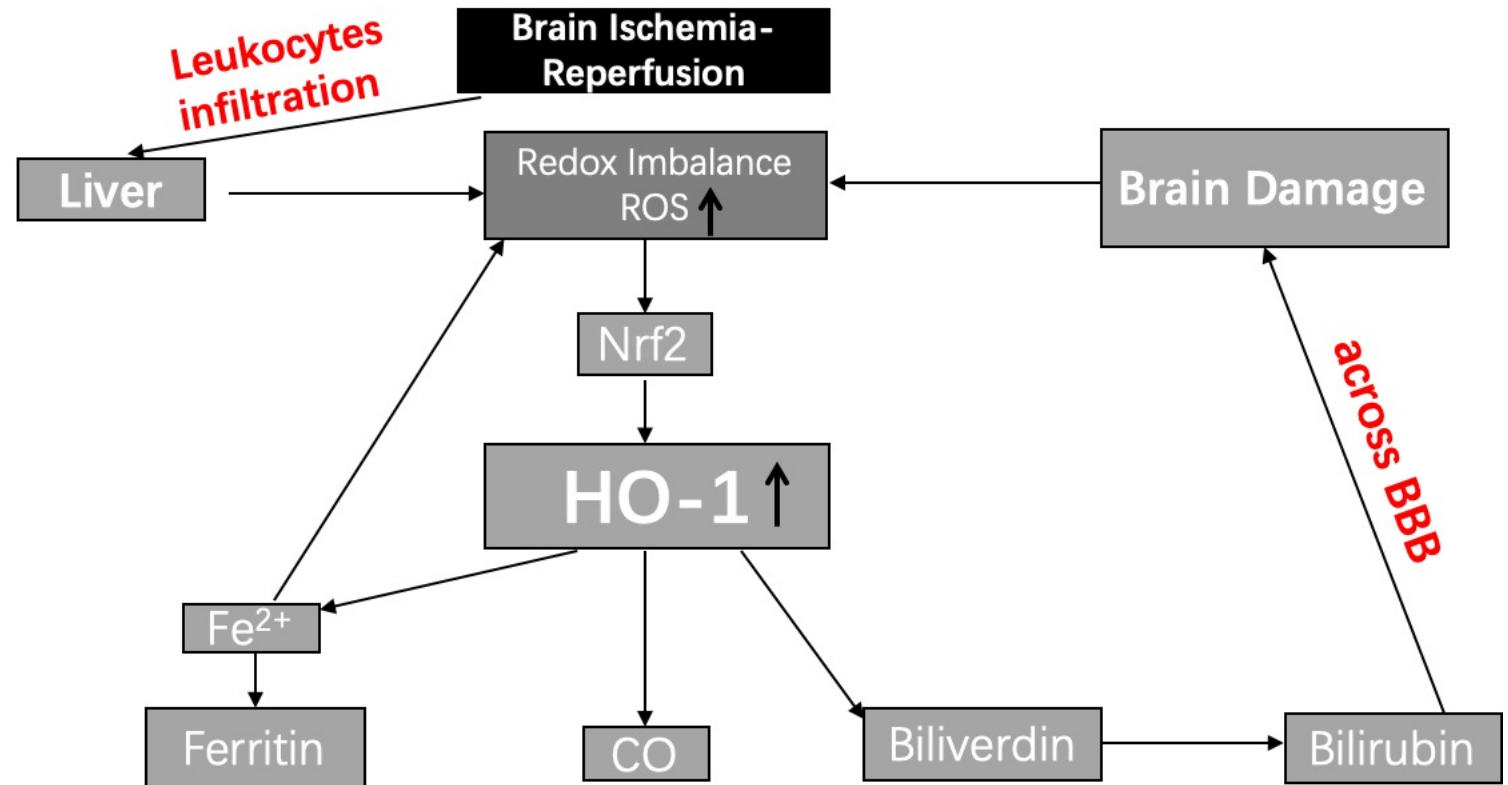

Supplement: Supplementary file 3 — Fig S3 [file CNS-28-226-s011.pdf]

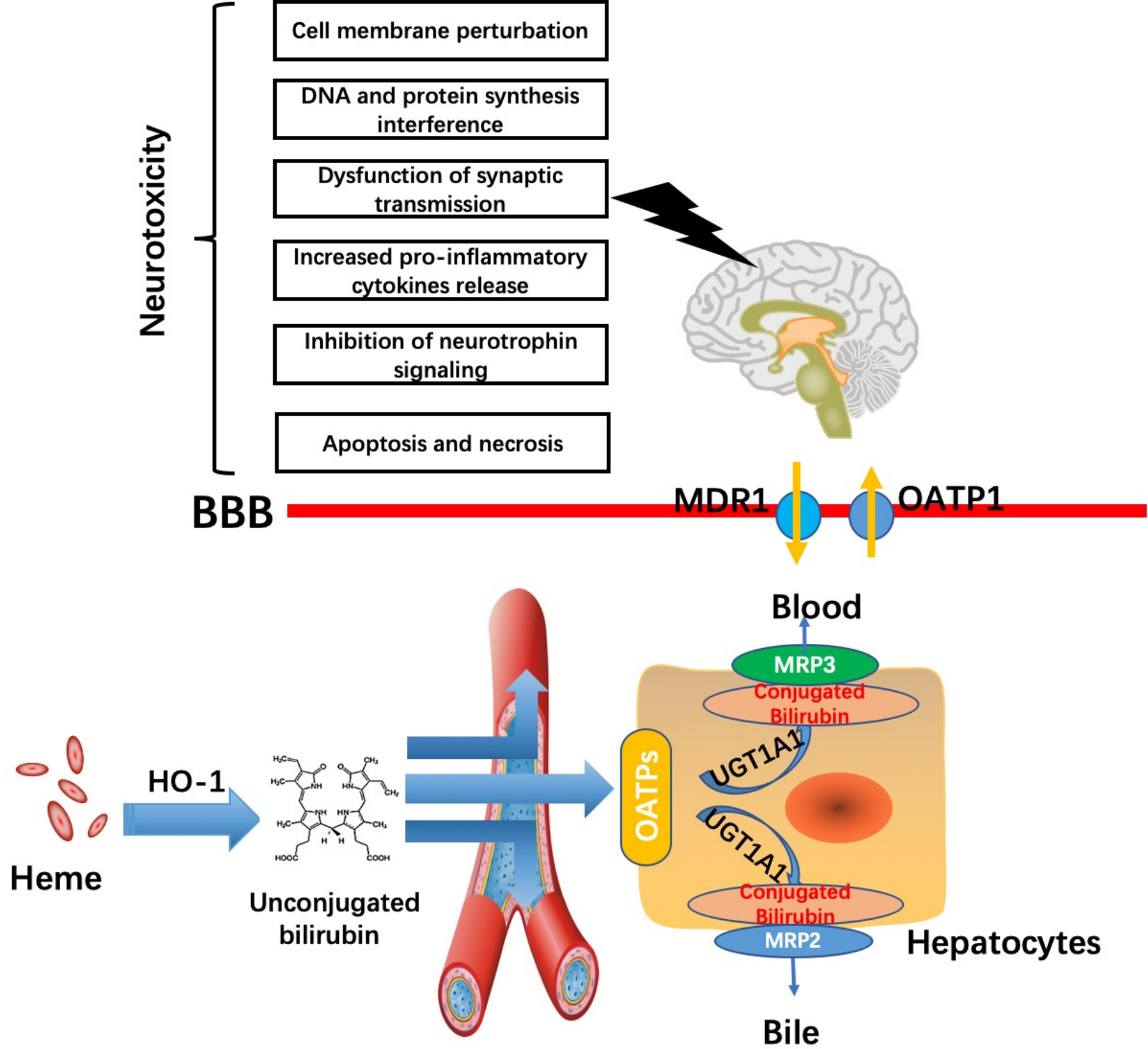

Supplement: Supplementary file 4 — Fig S4 [file CNS-28-226-s008.pdf]
