# Supplementary material for: Predictive value of different bilirubin subtypes for clinical outcomes in patients with acute ischemic stroke receiving thrombolysis therapy
Source: CNS Neurosci Ther. 2021 Nov 14;28(2):226–36. doi: 10.1111/cns.13759 (PMC8739039; doi:10.1111/cns.13759)
Supplement: Supplementary file 5 — Table S1 [file CNS-28-226-s012.docx]

| **Table S1** Baseline characteristics of participants across quartiles of serum total bilirubin levels | | | | | | |
| --- | --- | --- | --- | --- | --- | --- |
| **Characteristics** | **Total** | **Serum total bilirubin,μmol/L** | | | | ***P* Value for Trend** |
|  |  | **Q1 (<7.8)** | **Q2 (7.8-10.4)** | **Q3 (10.4-14.2)** | **Q4(≥14.2)** |  |
| **Patients, n** | 588 | 143 | 150 | 147 | 148 |  |
| **Age （year)** | 64.9±12.1 | 62.9±10.8 | 65.3±12.6 | 65.4±12.9 | 66.1±11.9 | 0.037* |
| **male，n (%)** | 389 (66.2) | 89 (62.2） | 101 (67.3） | 106 (72.1） | 93 (62.8） | 0.366 |
| **History of ischemic stroke** | 83 (14.1） | 18 (12.6） | 25 (16.7） | 20 (13.6） | 20 (13.5) | 0.379 |
| **History of intracerebral hemorrhage** | 15 (2.6） | 4 (2.8） | 2 (1.3) | 5 (3.4） | 4 (2.7) | 0.911 |
| **History of hypertension** | 372 (63.3） | 97 (67.8） | 89 (59.3) | 94 (63.9） | 92 (62.2) | 0.608 |
| **History of hyperlipemia** | 107 (18.2） | 34 (23.8） | 30 (20.0） | 24 (16.3） | 19 (12.8） | 0.027* |
| **History of diabetes mellitus** | 164 (27.9） | 38 (26.6） | 51 (34) | 37 (25.2） | 38 (25.7) | 0.745 |
| **Current cigarette smoking** | 205 (34.9) | 50 (35） | 55 (36.7) | 49 (33.3） | 51 (34.5) | 0.928 |
| **Current alcohol drinking** | 119 (20.2） | 30 (21） | 22 (14.7) | 32 (21.8） | 35 (23.6) | 0.884 |
| **Admission NIHSS score** | 4.0 (2.0-8.0) | 3.0 (1.0-6.0） | 3.5 (2.0-8.0） | 4.0 (2.0-8.0） | 4.0 (2.0-10.0） | 0.037* |
| **OTT, min** | 189.0 (139.0-250.0） | 184.0 (124.0-240.0） | 180.0 (130.0-249.3) | 192.7 (149.0-244.0） | 202.0 (142.0-258.8） | 0.046* |
| **Admission glucose, mmol/L** | 6.8 (5.4-8.7) | 7.6 (5.4-8.2） | 6.9 (5.8-8.9) | 6.9 (5.3-8.8） | 6.8 (5.3-8.8） | 0.343 |
| **Admission ALT, μmol/L** | 17.0 (12.0-24.0） | 17.0 (12.0-2.0） | 16.0 (12.0-24.0） | 18.0 (12.0-25.0） | 17.0 (12.0-25.0） | 0.303 |
| **Admission AST, μmol/L** | 20.0 (16.0-25.0） | 20.0 (16.0-23.0） | 19.0 (16.0-24.0） | 20.0 (17.0-26.0） | 20.0 (16.0-26.0） | 0.031* |
|  |  |  |  |  |  |  |
| Abbreviations: NIHSS: National Institutes of Health Stroke Scale; OTT: onset-to-treatment time; ALT: Alanine aminotransferase; AST: Aspartate aminotransferase | | | | | |  |
| **P*＜.05 |  |  |  |  |  |  |
