# Supplementary material for: Predictive value of different bilirubin subtypes for clinical outcomes in patients with acute ischemic stroke receiving thrombolysis therapy
Source: CNS Neurosci Ther. 2021 Nov 14;28(2):226–36. doi: 10.1111/cns.13759 (PMC8739039; doi:10.1111/cns.13759)
Supplement: Supplementary file 6 — Table S2 [file CNS-28-226-s009.docx]

| **Table S2** Baseline characteristics of participants across quartiles of serum indirect bilirubin levels | | | | | | |
| --- | --- | --- | --- | --- | --- | --- |
| **Characteristics** | **Total** | **Serum indirect bilirubin,μmol/L** | | | | ***P* Value for Trend** |
|  |  | **Q1 (<4.8)** | **Q2 (4.8-6.8)** | **Q3 (6.8-9.5)** | **Q4(≥9.5)** |  |
| **Patients, n** | 588 | 147 | 147 | 146 | 148 |  |
| **Age （year)** | 64.9±12.1 | 64.6±10.9 | 63.8±13.3 | 65.5±12.6 | 65.8±11.5 | 0.753 |
| **male，n (%)** | 388 (66.0） | 91 (61.9） | 96 (65.3） | 102 (69.9） | 99 (66.9） | 0.172 |
| **History of ischemic stroke** | 83 (14.1） | 16 (10.9) | 20 (13.6） | 23 (15.8) | 24 (16.2) | 0.189 |
| **History of intracerebral hemorrhage** | 15 (2.6） | 3 (2.0) | 2 (1.4） | 3 (2.1) | 7 (4.7) | 0.207 |
| **History of hypertension** | 355 (60.4） | 107 (72.8) | 84 (57.1） | 82 (56.2) | 82 (55.4) | 0.02* |
| **History of hyperlipemia** | 92 (15.6） | 36 (24.5) | 20 (13.6） | 18 (12.3) | 18 (12.2） | 0.005** |
| **History of diabetes mellitus** | 164 (27.9） | 43 (29.3) | 50 (34.0） | 38 (26.0) | 33 (22.3) | 0.871 |
| **Current cigarette smoking** | 205 (34.9） | 52 (35.4) | 55 (37.4） | 52 (35.6) | 46 (31.1) | 0.538 |
| **Current alcohol drinking** | 119 (20.2） | 32 (21.8) | 26 (17.7） | 29 (19.9) | 32 (21.6) | 0.885 |
| **Admission NIHSS score** | 4.0 (2.0-8.0） | 3.0 (1.0-6.0) | 4.0 (2.0-9.0） | 4.0 (1.8-8.0) | 4.0 (2.0-10.0) | 0.097 |
| **OTT, min** | 189.0 (139.0-250.0） | 185.0 (140.0-254.0) | 180.0 (125.0-250.0） | 196.0 (149.8-241.0) | 190.5 (140.0-253.0) | 0.444 |
| **Admission glucose, mmol/L** | 6.8 (5.4-8.7） | 6.6 (5.6-8.5) | 7.2 (5.8-9.4） | 6.9 (5.4-8.8) | 6.6 (4.9-8.5） | 0.694 |
| **Admission ALT, μmol/L** | 17.0 (12.0-24.0) | 16.0 (11.0-23.0) | 17.0 (12.0-24.0) | 16.5 (12.0-23.0) | 18.0 (13.0-25.7) | 0.046* |
| **Admission AST, μmol/L** | 20.0 (16.0-25.0) | 20.0 (16.0-23.0) | 19.0 (16.0-24.0) | 19.0 (16.0-24.25) | 20.0 (16.0-26.0) | 0.154 |
|  |  |  |  |  |  |  |
| Abbreviations: NIHSS: National Institutes of Health Stroke Scale; OTT: onset-to-treatment time; ALT: Alanine aminotransferase; AST: Aspartate aminotransferase | | | | | |  |
| **P*＜.05 |  |  |  |  |  |  |
| ***P*＜.01 |  |  |  |  |  |  |
