# Supplementary material for: Predictive value of different bilirubin subtypes for clinical outcomes in patients with acute ischemic stroke receiving thrombolysis therapy
Source: CNS Neurosci Ther. 2021 Nov 14;28(2):226–36. doi: 10.1111/cns.13759 (PMC8739039; doi:10.1111/cns.13759)
Supplement: Supplementary file 7 — Table S3 [file CNS-28-226-s004.docx]

| **Table S3** Odds ratios and 95% CI of moderate-severe brain edema for quartiles of each serum bilirubin pre-thrombolysis | | | |
| --- | --- | --- | --- |
|  |  | **Odds ratios (95% CI)** | |
| **Bilirubin types** | **No. of cases, n (%)** | **Model 1** | **Model 2** |
| **Total bilirubin** | 64 (10.9) | - | - |
| **Quartile 1** | 15 (10.5） | 1.00 (Ref.) | 1.00 (Ref.) |
| **Quartile 2** | 8 (5.3） | 0.512 (0.204-1.283） | 0.357 (0.116-1.098） |
| **Quartile 3** | 20 (13.6） | 1.353 (0.641-2.855） | 1.353 (0.543-3.376） |
| **Quartile 4** | 21 (14.2） | 1.637 (0.780-3.435） | 1.619 (0.643-4.078） |
| ***P* for trend** | - | 0.063 | 0.035* |
| **Each SD increase of log-total bilirubin** | - | 1.397 (1.116-1.749） | 1.478 (1.149-1.900） |
| **Indirect bilirubin** | 64 (10.9) | - | - |
| **Quartile 1** | 18 (12.2） | 1.00 (Ref.) | 1.00 (Ref.) |
| **Quartile 2** | 12 (8.2） | 0.682 (0.308-1.514） | 0.494 (0.190-1.286） |
| **Quartile 3** | 15 (10.3） | 0.823 (0.383-1.768） | 0.827 (0.326-2.095) |
| **Quartile 4** | 19 (12.8） | 1.043 (0.506-2.147） | 0.979 (0.401-2.391) |
| ***P* for trend** | - | 0.709 | 0.442 |
| **Each SD increase of log-indirect bilirubin** | - | 1.235 (0.991-1.538） | 1.328 (1.036-1.703） |
| **Direct bilirubin** | 64 (10.9) | - | - |
| **Quartile 1** | 11 (7.7） | 1.00 (Ref.) | 1.00 (Ref.) |
| **Quartile 2** | 14 (10.1） | 1.478 (0.629-3.472) | 1.627 (0.575-4.602) |
| **Quartile 3** | 12 (7.6） | 1.395 (0.564-3.449) | 1.526 (0.514-4.527) |
| **Quartile 4** | 27 (18.4） | 4.039 (1.809-9.015） | 4.483 (1.699-11.828) |
| ***P* for trend** | - | 0.001** | 0.008** |
| **Each SD increase of log-direct bilirubin** | - | 1.691 (1.334-2.144） | 1.691 (1.276-2.241） |
|  |  |  |  |
| **Model 1**: Adjusted for age, sex, onset-time to treatment, admission glucose, admission ALT, admission AST, current smoking, alcohol drinking, history of stroke, cerebral hemorrhage, hypertension, diabetes mellitus and hyperlipemia | | | |
|  |  |  |  |
| **Model 2**: Model 1+ admission NIHSS score | |  |  |
| **P*＜.05 |  |  |  |
| ***P*＜.01 |  |  |  |
