# Supplementary material for: Predictive value of different bilirubin subtypes for clinical outcomes in patients with acute ischemic stroke receiving thrombolysis therapy
Source: CNS Neurosci Ther. 2021 Nov 14;28(2):226–36. doi: 10.1111/cns.13759 (PMC8739039; doi:10.1111/cns.13759)
Supplement: Supplementary file 8 — Table S4 [file CNS-28-226-s005.docx]

| **Table S4** Odds ratios and 95% CI of sICH for quartiles of each serum bilirubin pre-thrombolysis | | | |
| --- | --- | --- | --- |
|  |  | **Odds ratios (95% CI)** | |
| **Bilirubin types** | **No. of cases, n (%)** | **Model 1** | **Model 2** |
| **Total bilirubin** | 35 (5.9) | - | - |
| **Quartile 1** | 9 (6.3） | 1.00 (Ref.) | 1.00 (Ref.) |
| **Quartile 2** | 5 (3.3） | 0.515 (0.164-1.619） | 0.381 (0.111-1.315） |
| **Quartile 3** | 8 (5.4） | 0.750 (0.271-2.073） | 0.725 (0.253-2.078） |
| **Quartile 4** | 13 (8.8） | 1.244 (0.496-3.120） | 1.119 (0.421-2.972） |
| ***P* for trend** | - | 0.404 | 0.303 |
| **Each SD increase of log-total bilirubin** | - | 1.159 (0.864-1.555） | 1.177 (0.861-1.610） |
| **Indirect bilirubin** | 35 (5.9) | - | - |
| **Quartile 1** | 10 (6.8） | 1.00 (Ref.) | 1.00 (Ref.) |
| **Quartile 2** | 8 (5.4） | 0.804 (0.299-2.162） | 0.717 (0.251-2.045） |
| **Quartile 3** | 6 (4.1） | 0.526 (0.179-1.549） | 0.561 (0.182-1.732） |
| **Quartile 4** | 11 (7.4） | 0.928 (0.369-2.335） | 0.906 (0.343-2.398） |
| ***P* for trend** | - | 0.669 | 0.745 |
| **Each SD increase of log-indirect bilirubin** | - | 1.090 (0.812-1.464） | 1.116 (0.816-1.527） |
| **Direct bilirubin** | 35 (5.9) | - | - |
| **Quartile 1** | 6 (4.2） | 1.00 (Ref.) | 1.00 (Ref.) |
| **Quartile 2** | 7 (5.0） | 1.162 (0.372-3.628) | 1.016 (0.324-3.474) |
| **Quartile 3** | 5 (3.2） | 0.793 (0.225-2.792) | 0.704 (0.189-2.627) |
| **Quartile 4** | 17 (11.6） | 2.907 (1.062-7.957) | 2.549 (0.897-7.242) |
| ***P* for trend** | - | 0.029* | 0.058 |
| **Each SD increase of log-direct bilirubin** | - | 1.611 (1.117-2.323) | 1.555 (1.057-2.287) |
|  |  |  |  |
| **Model 1**: Adjusted for age, sex, onset-time to treatment, admission glucose, admission ALT, admission AST, current smoking, alcohol drinking, history of stroke, cerebral hemorrhage, hypertension, diabetes mellitus and hyperlipemia | | | |
|  |  |  |  |
| **Model 2**: Model 1+ admission NIHSS score | |  |  |
| **P*＜.05 |  |  |  |
