# Supplementary material for: Predictive value of different bilirubin subtypes for clinical outcomes in patients with acute ischemic stroke receiving thrombolysis therapy
Source: CNS Neurosci Ther. 2021 Nov 14;28(2):226–36. doi: 10.1111/cns.13759 (PMC8739039; doi:10.1111/cns.13759)
Supplement: Supplementary file 9 — Table S5 [file CNS-28-226-s006.docx]

| **Table S5** Discriminative power comparison of bilirubin subtypes pre-thrombolysis for clinical outcomes | | | | | |
| --- | --- | --- | --- | --- | --- |
| **Clinical outcomes** | | | **C-statistic** | **95% CI** | ***P* value** |
| **3-month death and major disability** | | |  |  |  |
| DBIL | | | 0.622 | 0.569-0.675 | - |
| TBIL | | | 0.585 | 0.530-0.639 | 0.036* |
| IBIL | | | 0.548 | 0.493-0.603 | 0.004** |
| **3-month death** | | |  |  |  |
| DBIL | | | 0.648 | 0.567-0.729 | - |
| TBIL | | | 0.591 | 0.501-0.681 | 0.019* |
| IBIL | | | 0.552 | 0.462-0.642 | 0.005** |
| **moderate-severe edema** | | |  |  |  |
| DBIL | | | 0.640 | 0.562-0.718 | - |
| TBIL | | | 0.593 | 0.512-0.674 | 0.046* |
| IBIL | | | 0.549 | 0.467-0.631 | 0.01* |
|  |  |  |  |  |  |
| **P*＜.05 |  |  |  |  |  |
| ***P*＜.01 |  |  |  |  |  |
