# Supplementary material for: Predictive value of different bilirubin subtypes for clinical outcomes in patients with acute ischemic stroke receiving thrombolysis therapy
Source: CNS Neurosci Ther. 2021 Nov 14;28(2):226–36. doi: 10.1111/cns.13759 (PMC8739039; doi:10.1111/cns.13759)
Supplement: Supplementary file 10 — Table S6 [file CNS-28-226-s002.docx]

| **Table S6** Incremental predictive value of different bilirubin subtypes for 3-month mortality | | | | | | | |
| --- | --- | --- | --- | --- | --- | --- | --- |
|  | **Discrimination** | |  | **Reclassification** | | | |
|  | **C-statistic (95% CI)** | ***P* value** |  | **NRI (95% CI)** | ***P* value** | **IDI (95% CI)** | ***P* value** |
| **CM** | 0.830 (0.794-0.862) | - |  | 1.00 (Ref.) | - | 1.00 (Ref.) | - |
| **CM + TBIL** | 0.829 (0.794-0.861) | 0.965 |  | 0.285 (-0.037-0.607) | 0.082 | 0.001 (-0.003-0.005) | 0.607 |
| **CM + IBIL** | 0.828 (0.792-0.860) | 0.865 |  | 0.389 (0.069-0.709) | 0.071 | 0.001 (-0.002-0.003) | 0.789 |
| **CM + DBIL** | 0.836 (0.801-0.868) | 0.577 |  | 0.331 (0.009-0.652) | 0.044* | 0.005 (0.004-0.013) | 0.016* |
|  |  |  |  |  |  |  |  |
| **CM**: age, sex, onset-time to treatment, admission NIHSS score, admission glucose, admission ALT, admission AST, current smoking, alcohol drinking, history of stroke, cerebral hemorrhage, hypertension, diabetes mellitus and hyperlipemia | | | | | | | |
|  |  |  |  |  |  |  |  |
| **P*＜.05 |  |  |  |  |  |  |  |
