# Supplementary material for: Predictive value of different bilirubin subtypes for clinical outcomes in patients with acute ischemic stroke receiving thrombolysis therapy
Source: CNS Neurosci Ther. 2021 Nov 14;28(2):226–36. doi: 10.1111/cns.13759 (PMC8739039; doi:10.1111/cns.13759)
Supplement: Supplementary file 11 — Table S7 [file CNS-28-226-s007.docx]

| **Table S7** Incremental predictive value of different bilirubin subtypes for moderate-severe cerebral edema | | | | | | | |
| --- | --- | --- | --- | --- | --- | --- | --- |
|  | **Discrimination** | |  | **Reclassification** | | | |
|  | **C-statistic (95% CI)** | ***P* value** |  | **NRI (95% CI)** | ***P* value** | **IDI (95% CI)** | ***P* value** |
| **CM** | 0.900 (0.869-0.926) | - |  | 1.00 (Ref.) | - | 1.00 (Ref.) | - |
| **CM + TBIL** | 0.905 (0.874-0.930) | 0.539 |  | 0.241 (-0.014-0.495) | 0.063 | 0.022 (-0.003-0.047) | 0.083 |
| **CM + IBIL** | 0.904 (0.874-0.929) | 0.526 |  | 0.162 (-0.093-0.418) | 0.214 | 0.011 (-0.006-0.028) | 0.200 |
| **CM + DBIL** | 0.909 (0.879-0.934) | 0.292 |  | 0.386 (0.128-0.6445) | 0.003** | 0.038 (0.004-0.072) | 0.028* |
|  |  |  |  |  |  |  |  |
| **CM**: age, sex, onset-time to treatment, admission NIHSS score, admission glucose, admission ALT, admission AST, current smoking, alcohol drinking, history of stroke, cerebral hemorrhage, hypertension, diabetes mellitus and hyperlipemia | | | | | | | |
|  |  |  |  |  |  |  |  |
| **P*＜.05 |  |  |  |  |  |  |  |
| ***P*＜.01 |  |  |  |  |  |  |  |
