# Supplementary material for: Predictive value of different bilirubin subtypes for clinical outcomes in patients with acute ischemic stroke receiving thrombolysis therapy
Source: CNS Neurosci Ther. 2021 Nov 14;28(2):226–36. doi: 10.1111/cns.13759 (PMC8739039; doi:10.1111/cns.13759)
Supplement: Supplementary file 12 — Table S8 [file CNS-28-226-s003.docx]

| **Table S8** Odds ratios and 95% CI of primary outcome for quartiles of serum direct bilirubin | | | |
| --- | --- | --- | --- |
|  |  | **Odds ratios (95% CI)** | |
|  | **No. of cases, n (%)** | **Model 1** | **Model 2** |
| **Direct bilirubin** | 99 (17.1) | - | - |
| **Quartile 1** | 24 (17.6) | 1.00 (Ref.) | 1.00 (Ref.) |
| **Quartile 2** | 34 (24.1) | 1.174 (0.948-3.321) | 1.935 (0.921-4.066) |
| **Quartile 3** | 38 (24.7) | 1.701 (0.912-3.172) | 1.547 (0.744-3.217) |
| **Quartile 4** | 53 (35.8) | 2.746 (1.496-5.039) | 2.416 (1.184-4.930) |
| ***P* for trend** | - | 0.013* | 0.039* |
| **Each SD increase of log-total bilirubin** | - | 1.582 (1.254-1.996) | 1.562 (1.184-2.061) |
|  |  |  |  |
| **Direct bilirubin (μmol/L)**: Quartile 1:＜9.8, Quartile 2:9.8-13.2, Quartile 3: 13.2-17.7, Quartile 4: ≥17.7 | | | |
| **Model 1**: Adjusted for age, sex, onset-time to treatment, admission glucose, admission ALT, admission AST, current smoking, alcohol drinking, history of stroke, cerebral hemorrhage, hypertension, diabetes mellitus and hyperlipemia | | | |
|  |  |  |  |
| **Model 2**: Model 1+ admission NIHSS score | |  |  |
| **P*＜.05 |  |  |  |
