# Supplementary material for: Predictive value of different bilirubin subtypes for clinical outcomes in patients with acute ischemic stroke receiving thrombolysis therapy
Source: CNS Neurosci Ther. 2021 Nov 14;28(2):226–36. doi: 10.1111/cns.13759 (PMC8739039; doi:10.1111/cns.13759)
Supplement: Supplementary file 13 — Table S9 [file CNS-28-226-s013.docx]

| **Table S9** Incremental predictive value of serum direct bilirubin for primary outcome | | | | | | | |
| --- | --- | --- | --- | --- | --- | --- | --- |
|  | **Discrimination** | |  | **Reclassification** | | | |
|  | **C-statistic (95% CI)** | ***P* value** |  | **NRI (95% CI)** | ***P* value** | **IDI (95% CI)** | ***P* value** |
| **CM** | 0.825 (0.789-0.857) | - |  | 1.00 (Ref.) | - | 1.00 (Ref.) | - |
| **CM + DBIL** | 0.843 (0.807-0.874) | 0.566 |  | 0.259 (0.066-0.453) | 0.009** | 0.025 (0.008-0.043) | 0.005** |
|  |  |  |  |  |  |  |  |
| **CM**: age, sex, onset-time to treatment, admission NIHSS score, admission glucose, admission ALT, admission AST, current smoking, alcohol drinking, history of stroke, cerebral hemorrhage, hypertension, diabetes mellitus and hyperlipemia | | | | | | | |
|  |  |  |  |  |  |  |  |
| ***P*＜.01 |  |  |  |  |  |  |  |
